# Single‐Cell Atlas Reveals Tumorigenic Profiles and Immune Dynamics of Adrenal Incidentalomas

**DOI:** 10.1002/advs.202413493

**Published:** 2025-04-07

**Authors:** Meng Wang, Guangmin Zheng, Xiaoyong Hu, Feng Tian, Tuo Li, Zheng Zhang, Kan Gong, Shiwei Chen, Lin Yuan, Yu Qi, Lin Li, Daofu Cheng, Liu Liu, Fuqiang Liu, Yujing Sun, Xiangdong Fang, Ruxing Zhao, Bing Liu, Chao Zhang

**Affiliations:** ^1^ Department of Orthopedics and Precision Research Center for Refractory Diseases Shanghai General Hospital Shanghai Jiao Tong University School of Medicine Shanghai 200080 China; ^2^ Department of Endocrinology Songjiang Research Institute Shanghai Key Laboratory of Emotions and Affective Disorders (LEAD) Songjiang Hospital Affiliated to Shanghai Jiao Tong University School of Medicine Shanghai 201600 China; ^3^ CAS Key Laboratory of Genome Sciences and Information Beijing Institute of Genomics Chinese Academy of Sciences/China National Center for Bioinformation Beijing 100101 China; ^4^ Department of Urology Shanghai Jiao Tong University Affiliated Sixth People's Hospital Shanghai 200000 China; ^5^ Hebei Key Laboratory of Medical Data Science Institute of Biomedical Informatics School of Medicine Hebei University of Engineering Handan Hebei Province 056009 China; ^6^ Department of Endocrinology Shanghai Changzheng Hospital Shanghai 200003 China; ^7^ Department of Biochemistry and Molecular Biology School of Basic Medical Sciences Department of Urology Peking University First Hospital Peking University Health Science Center Beijing 100035 China; ^8^ Department of Pathology Shanghai General Hospital Shanghai Jiao Tong University School of Medicine Shanghai 201620 China; ^9^ Department of Urology Eastern Hepatobiliary Surgery Hospital Shanghai 201805 China; ^10^ Shanghai Yuhui Pharmaceutical Technology (Group) Co., Ltd. Shanghai 201203 China; ^11^ Department of Endocrinology and Metabolism Qilu Hospital of Shandong University Shandong Provincial Key Laboratory of Spatiotemporal Regulation and Precision Intervention in Endocrine and Metabolic Diseases Shandong University Jinan 250012 China

**Keywords:** adrenal incidentaloma, clusterin, imaging, immune microenvironment, scRNA‐seq, SF1

## Abstract

Adrenal incidentalomas (AIs) are commonly detected endocrine lesions, identified during imaging for unrelated conditions. These lesions exhibit considerable heterogeneity and diverse clinical outcomes. This study employed single‐cell RNA sequencing to investigate tumorigenic characteristics of AIs, including non‐functional adrenocortical adenomas, Conn's syndrome, and pheochromocytomas. Through integrating public datasets, 302 696 cells are analyzed. Three adrenocortical cell subtypes exhibit gene expression patterns linked to tumorigenesis. Clusterin emerges as a potential biomarker for adrenocortical adenomas. Adrenocortical tumor cells show dysregulated hormone secretion and transcription factor steroidogenic factor 1 (SF1) is significantly upregulated, distinguishing cortical from medullary tumors. In pheochromocytomas, a MYCN proto‐oncogene (MYCN)‐positive cluster correlates with poorer survival. Immune microenvironment analysis reveals specific immune subtypes and roles in tumor progression. Specifically, myeloid cells may regulate benign tumors, while lymphoid cells, such as CD8‐positive (CD8+) T cells, appear to promote immune activation and infiltration in malignant tumors. Overall, this study enhances the understanding of adrenal adenoma heterogeneity, revealing crucial transcriptional profiles, immune interactions, and clinically relevant candidate biomarkers.

## Graphical abstract

Single‐cell RNA sequencing reveals cellular heterogeneity in adrenal incidentalomas, identifying distinct tumor cell populations. Clusterin is recognized as a biomarker for adrenocortical tumors, correlating with established markers. MYCN‐positive clusters in pheochromocytomas indicated poorer survival. Immune microenvironment analysis shows significant tumor‐immune interactions. This comprehensive study of 302 696 cells offers novel insights to improve adrenal tumor diagnosis and treatment.

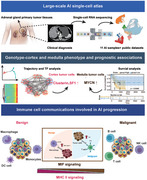

## 1 Introduction

Adrenal incidentalomas (AI) are prevalent endocrine lesions that were incidentally discovered during imaging performed for other reasons.^[^ ^1^ ^]^ ≈6% of adults are affected by AI, and their incidence is rising due to the widespread application of advanced imaging techniques, technological improvements, and an aging population.^[^ ^2^ ^]^ The classification of AI patients is based on both etiology and endocrine activity. The etiology includes tumors originating from the adrenal cortex and medulla, with the majority being of cortical origin. Functioning AIs are characterized by excessive hormonal secretion, such as cortisol, aldosterone, or androgens, leading to conditions such as Cushing's syndrome, Conn's syndrome, or virilization, respectively. Conversely, non‐functioning AI does not exhibit any hormonal activity.^[^ ^1^ ^]^ Adrenocortical AIs are predominantly benign.^[^ ^3^ ^]^ Notably, malignant AIs, including adrenocortical carcinoma (ACC), are less frequent and only account for 0.2% of all cancers.^[^ ^4^ ^]^ Individualized treatment strategies based on imaging findings, endocrine assessments, and clinical symptoms are essential for managing each AI patient. Advances in surgical techniques have enhanced outcomes for patients with functional adrenal lesions, such as adrenocortical adenomas (ACAs) and pheochromocytomas (PCCs), which often require excision. However, the prognosis for AI patients remains challenging due to the heterogeneous nature of these tumors and the lack of specific biomarkers.^[^ ^5^ ^]^ Additionally, diagnostic challenges persist due to substantial variability in technical and methodological approaches. Therefore, further research is warranted to identify novel and reliable biomarkers for early diagnosis and to devise more effective therapeutic strategies with the aim of improving patient outcomes.

Recent advancements in single‐cell RNA sequencing (scRNA‐seq) technology have greatly enhanced our capacity to investigate the complex hormone secretion profiles, pathogenesis, and tumor microenvironment (TME) of adrenal tumors. Notably, neuroblastoma (NB) has been a primary focus in the application of scRNA‐seq to adrenal tumors.^[^ ^6^, ^7^, ^8^, ^9^, ^10^ ^]^ Investigations utilizing scRNA‐seq in NB have revealed distinct cellular subpopulations within the tumor that contribute to its aggressive behavior and differential therapeutic responses. These studies have identified key transcriptional signatures linked to tumor progression, such as the upregulation of genes involved in neural crest differentiation, survival pathways, and stress responses. Furthermore, scRNA‐seq has provided insights into the TME of NB, highlighting the significant roles of infiltrating immune cells, endothelial cells, and stromal components in influencing the tumor's response to therapy and its metastatic potential. Although neuroblastoma has been extensively studied using single‐cell approaches, other adrenal tumors, such as pheochromocytomas and aldosterone‐producing adenomas, have received comparatively less attention. Recent scRNA‐seq studies of underexplored tumor types have begun to reveal their distinct cellular characteristics, highlighting the significant role of hormone‐secreting cell populations in tumor progression.^[^ ^11^, ^12^, ^13^ ^]^ Additionally, scRNA‐seq analysis of pheochromocytomas has identified immune infiltration patterns within the TME, which may influence both tumor progression and patient prognosis.^[^ ^13^ ^]^ While these studies have provided valuable insights, the limited sample sizes and representation of AI subtypes have highlighted the need for further research to achieve a more comprehensive understanding of AI heterogeneity and its potential implications for treatment strategies and patient survival outcomes. This study aims to build on these findings by expanding the theoretical framework and broadening the scope of AI subtype analysis.

To address the aforementioned challenges, this study presents a comprehensive, organ‐specific scRNA‐seq atlas of adrenal tumors. The atlas includes 11 primary AI patient samples. Additionally, publicly available single‐cell datasets were incorporated, comprising 2 fetal adrenal controls, 15 NB samples, and 4 PCC samples with their peritumoral tissues. By utilizing this diverse and unbiased sample collection, patients were stratified, and disease‐specific markers for different adrenal tumor subtypes were identified. Moreover, to further elucidate the factors influencing hormone secretion and the inter‐tumoral microenvironment in AIs, bulk RNA‐seq data, and multiplexed immunofluorescence data were also integrated. Although the majority of the AI samples were benign, this study represents the largest single‐cell AI sampling effort to date, encompassing a broad spectrum of AI characteristics.

## 2 Results

### 2.1 Global Atlas of Heterogenous Human Adrenal Tumors Cells

**Figure 1:**
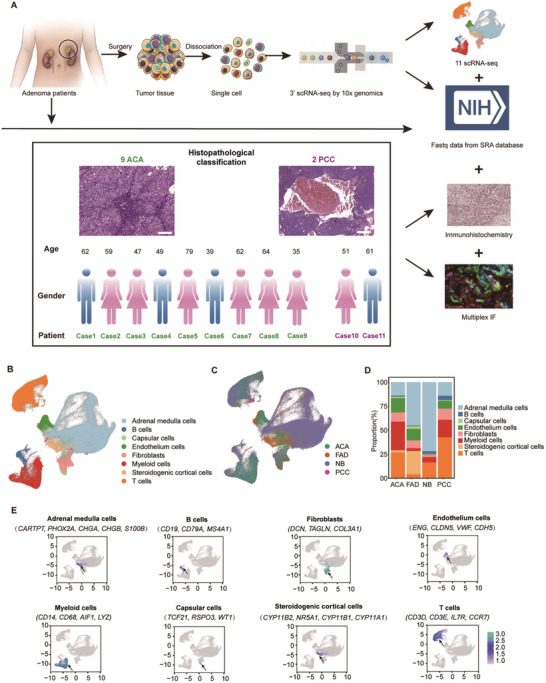
Single‐cell Profiling of Integrated Human Adrenal Gland, Neuroblastoma and Incidentaloma. A) Schematic of the experimental design and workflow: surgically resected adrenal tumor tissues from 11 patients (9 ACAs and 2 PCCs) were mechanically and enzymatically dissociated into single cells, scRNA‐seq analysis was performed, infiltration was validated using immunohistochemistry, and publicly available scRNA‐seq datasets were integrated for combined analysis. B) UMAP plot of adrenal tumor cellular composition, revealing eight distinct clusters: steroidogenic cortical cells, B cells, fibroblasts, endothelium, myeloid cells, capsular cells, adrenal medulla cells, and T cells. C) UMAP of 302696 cells from ACAs, PCCs, fetal adrenal samples (FAD), and NB samples; colored by sample origin. D) Stacked bar plot showing the proportion of cell types across different adrenal gland or tumor types (ACAs, FAD, NB, and PCCs). Colors correspond to cell type annotations in B). E) Representative marker expression (log‐normalized) of the eight identified cell clusters. Each subplot represents the relative staining intensity of key markers.

To investigate the cellular and molecular characteristics of different subtypes of human AIs, scRNA‐seq was performed on fresh surgically resected tumor specimens collected from 11 AI patients. Histopathological analysis classified 9 of these cases as adrenocortical adenomas (ACAs) and 2 as pheochromocytomas (PCCs) (**Figure** **1**A; Table S1, Supporting Information). The identified features of the majority of AIs were further confirmed with contrast (enhanced) CT imaging (Figure S1, Supporting Information). Of these 11 patients, 7 were female, and 4 were male. Furthermore, 9 of these participants were aged >40 years. Out of the 87163 sequenced cells, 62784 (72%) passed the quality threshold, with an average of ≈32647 reads per cell alignment (see STAR methods; Table S2, Supporting Information). The study incorporated scRNA‐seq data from 2 fetal adrenal samples to serve as normal controls and 15 neuroblastoma (NB) samples, both sourced from the GSE137804 dataset and 4 public PCCs’ single‐cell data with the peritumoral tissues,^[^ ^14^ ^]^ to construct a more comprehensive single‐cell atlas of adrenal tumors (Figure 1B). Table S2 (Supporting Information) summarizes information and key study features of all the samples. Overall, this core adrenal tumor atlas was constructed by curating, quality‐assuring, and pre‐analyzing transcriptomic data from publicly available studies and our own AI dataset, employing two high‐throughput single‐cell transcriptome sequencing platforms (see STAR methods; Figure S2A; Table S2, Supporting Information).

Furthermore, this atlas was annotated with eight principal cell types, defined based on previously established canonical single‐cell signatures. These included adrenal medulla cells, B cells, capsular cells, endothelium cells, fibroblasts, myeloid cells, steroidogenic cortical cells, and T cells (Figure 1B; Figure S2C,D, Supporting Information). The cellular composition of each AI type within the core atlas is illustrated in Figure 1C,D, and all eight cell types were consistently identified across AI patients (Figure S2B, Supporting Information). Distinctly, adrenal medulla cells were characterized by the expression of CARTPT, PHOX2A, CHGA, CHGB, and S100B, while adrenal steroidogenic cortical cells exhibited cortical marker expression, including CYP11B2, NR5A1, CYP11B1, and CYP11A1 (Figure 1E). To delineate AIs tumor cells from heterogeneous populations, chromosomal gene expression patterns were analyzed (refer to STAR Methods for additional details) (Figure S3A, Supporting Information). Chromosomal copy number variation (CNV) across specific genomic regions was quantified using a two‐component Gaussian mixture model (Figure S3B, Supporting Information). Tumor cells demonstrate increased genomic instability, which aligns with the identified divergence in genomic profiles between cortical and medullary regions in adrenocortical tumors.

### 2.2 Comparison Between Normal and Tumor Cells Reveals Altered Secretory Functions of Cortical Tumor Cells

**Figure 2:**
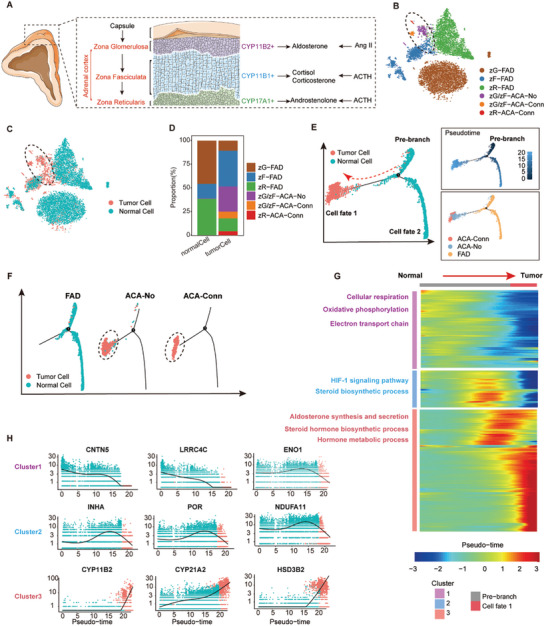
Altered Secretory Functions of Steroidogenic Cortical Tumor Cells. A) Schematic diagram showing the structure of the human adrenal cortex and its three distinct layers: zG (zona glomerulosa), zF (zona fasciculata), and zR (zona reticularis). Key markers for each layer include CYP11B2 (zG), CYP11B1 (zF), and CYP17A1 (zR), which are associated with aldosterone, cortisol, and androgen biosynthesis pathways, respectively. B) t‐SNE visualization showing detailed annotation of adrenocortical cell subpopulations, grouped by three adrenal cortex layers and tumor cells. B) Clusters grouped by cortex layers and sample origin. The clusters represent zG‐FAD, zF‐FAD, and zR‐FAD (zona glomerulosa, fasciculata, and reticularis in fetal adrenal), zG/zF‐ACA‐No and zG/zF‐ACA‐Conn (zona glomerulosa and fasciculata in no‐functional adrenocortical adenomas or Conn's syndrome), and zR‐ACA‐Conn (zona reticularis in Conn's syndrome). C) Tumor cell‐specific distribution among adrenocortical clusters, with circles highlighting the main tumor cell populations. D) Frequency distribution of different adrenocortical cell types among normal and tumor cells. E) Trajectory inference of normal and tumor cells using Monocle2. (Left) Trajectories in a two‐dimensional space representing normal (blue) and tumor (red) cells. Arrows indicate pseudotime progression. (Right) Trajectory plots separated by tumor types, with cells colored by inferred cell state (top) or tumor sample type (bottom). F) Pseudotime trajectory plots showing the distribution of cells in FAD, ACA‐No, and ACA‐Conn groups. Tumor cells are circled for clarity. G) Heatmap of differentially expressed genes clustered hierarchically across pseudotime. Columns represent individual cells (ordered by pseudotime), and rows represent gene expression (z‐scores). The representative gene functions and GO pathways of each profile were shown. H) Line plots showing the top 3 gene signatures from each cluster in G) along pseudotime. Dashed lines represent natural cubic splines fit for individual cells.

The adrenal cortex plays an important role in maintaining the balance of paracrine and endocrine signals (**Figure** **2**A). Adrenocortical incidentalomas develop as a consequence of functional abnormalities within cortical cells.^[^ ^15^ ^]^ To achieve a comprehensive understanding of the cellular states and developmental trajectories associated with adrenocortical tumors, a second round of clustering specifically targeting the steroidogenic cortical cells was conducted (Figure 2B; Figure S4A, Supporting Information). This analysis was informed by established markers for the three cortical layers described in the literature (zG, zona glomerulosa: DACH1, VSNL1; zF, zona fasciculata: CCN3, NCAM1; zR, zona reticularis: CYB5A, SULT2A1).^[^ ^16^ ^]^ InferCNV in Figure S3 (Supporting Information) revealed a significant clustering of tumor cells within the three pathological adrenal cortical subtypes: zG/zF‐ACA‐No, zG/zF‐ACA‐Conn, and zR‐ACA‐Conn. Notably, zG/zF‐ACA‐No and zG/zF‐ACA‐Conn were distinguished by their expression of zG markers while concurrently exhibiting features characteristic of the zG, including the expression of steroidogenic genes such as CYB21A1, CYP11B2, and HSD3B2 (Figure S4A, Supporting Information).^[^ ^17^, ^18^ ^]^ This finding suggests the presence of either functional convergence or adaptive alterations in tumor cells spanning multiple cortical zones. Furthermore, these subtypes may represent atypical differentiation states, underscoring the intricate organization of adrenal cortical tumor tissues.

To understand the cell fate bias of normal cells toward tumor cells in different tumors, Monocle2 was employed to reconstruct the trajectory of cellular differentiation states and estimate the probabilities of cell fate transitions. The analysis uncovered a progressive commitment of normal cells toward tumorigenic states (Figure 2E,F; Figure S4E, Supporting Information). In fetal samples, normal cells were primarily located in the pre‐branch and cell fate 2 regions. Benign tumor cells, those from ACA samples, were predominantly positioned in cell fate 1. Furthermore, the distribution of cortical subtypes along the three fate branches was evaluated (Figure S4F, Supporting Information). Importantly, the three pathological adrenal cortical subtypes demonstrated elevated expression levels of genes linked to an increased risk of adrenocortical diseases^[^ ^19^ ^]^ (Figure S4G,H, Supporting Information).

To investigate transcriptomic alterations during tumorigenesis, pseudo‐time analysis, and Gene Ontology (GO) annotations were applied to evaluate changes in gene expression patterns and associated biological processes (Figure 2G; Table S4, Supporting Information). Marker genes were identified within each of the three cell clusters, representing the progression from normal to tumor cells, with their expression levels exhibiting gradual and dynamic shifts (Figure 2G,H). Notably, several key genes involved in steroid synthesis, including CYP11B2, CYP21A2, and HSD3B2, were significantly upregulated along the trajectories toward the tumor cell lineage. This upregulation aligns with GO term enrichment for disrupted aldosterone synthesis and secretion, steroid hormone biosynthesis, and hormone metabolic process in tumor cells. Additionally, changes in two key pathways—the HIF‐1 signaling pathway and the steroid biosynthetic process—were detected during the transition of cortical cells from a normal to a tumorigenic phenotype (Figure 2G). The HIF‐1 signaling pathway (hypoxia‐inducible factor‐1 signaling pathway) was notably activated during tumor progression.^[^ ^20^ ^]^ This activation is likely associated with tumor cell adaptation to the microenvironment, particularly under hypoxic conditions,^[^ ^21^ ^]^ as well as metabolic reprogramming, enabling enhanced survival and proliferation of tumor cells. These findings suggest that adrenal cortical tumor cells undergo adaptive metabolic adjustments to accommodate aberrant metabolic demands.

### 2.3 Identification of CLU as a Potential Marker in Adrenocortical Incidentalomas

**Figure 3:**
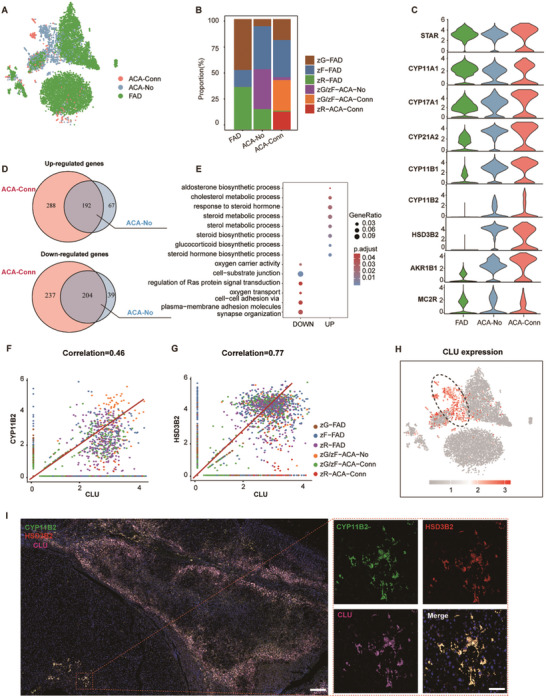
The Landscape of Tumor Heterogeneity in Adrenocortical Adenomas. A) t‐SNE plot showing annotated adrenocortical cell populations from different tumors, with colors and labels indicating patient types. B) Stacked bar plot representing the frequency distribution of ACAs across six distinct adrenocortical cell subpopulations. C) Violin plot displaying the expression of steroidogenic enzymes (STAR, CYP11A1, CYP17A1, CYP21A2, CYP11B1, CYP11B2, AKR1B1, and HSD3B2) and MC2R in FAD, ACA‐No, and ACA‐Conn groups. D) Venn diagrams showing the overlap of f upregulated genes (top) and downregulated genes (bottom) in ACA‐Conn compared to ACA‐No groups. E) GO Functional enrichment analysis of the upregulated and downregulated genes in the shared gene clusters identified in D). Pathways are ranked by adjusted *p*‐values (p.adjust), with p.adjust ≤ 0.05 considered significant. F,G) Pearson correlation analysis showing the relationship between CLU expression and CYP11B2 (G, correlation = 0.46) or HSD3B2 (H, correlation = 0.77) across six cell clusters from ACAs. H) t‐SNE plot showing CLU expression in ACA samples. I) Immunofluorescence staining of ACAs for CYP11B2 (green), HSD3B2 (red), CLU (pink), and DAPI (nuclei, blue). Scale bars = 100 µm.

Based on clinical evaluations, nine ACAs were included in this study and divided into two groups: Conn's syndrome and non‐functional tumors (Table S1, Supporting Information). The observed heterogeneity in the distribution of cortical cells within these samples led to a detailed investigation of their cellular composition and properties (**Figure** **3**A,B). Adrenal steroidogenesis involves a series of enzymatic reactions that convert cholesterol into biologically active steroid hormones in response to angiotensin II, adrenocorticotropic hormone (ACTH), and other peptide hormones.^[^ ^22^ ^]^ Previous clinical and molecular research has highlighted the involvement of steroidogenic pathway enzymes, including STAR, CYP11A1, CYP17A1, CYP21A2, CYP17B1, CYP17B2, AKR1B1, and HSD3B2,^[^ ^23^, ^24^ ^]^ as well as the melanocortin receptor 2 (MC2R, the ACTH receptor),^[^ ^25^ ^]^ in the pathogenesis of functional adrenocortical tumors. To explore the relationship between these endocrine disruptors and the disease phenotypes observed in this study, the expression of steroidogenic genes and MC2R were analyzed in ACAs. (Figure 3C). The findings indicated that the aldosterone‐producing enzymes HSD3B2 and CYP11B2 were both upregulated in Conn's syndrome and non‐functional ACAs, suggesting that hormone synthesis and secretion abnormalities are associated with ACAs.

A differential gene expression analysis was subsequently performed to compare Conn's syndrome with non‐functional adrenocortical tumors (Figure S5A–C; Table S5, Supporting Information). In line with the observations in Figure 3C, multiple genes associated with hormone biosynthesis, including CYP11B2 and AKR1B1, exhibited significant upregulation in Conn's syndrome (Figure S5B, Supporting Information). Furthermore, a total of 192 upregulated and 204 downregulated genes were identified across samples from both Conn's syndrome and non‐functional adrenocortical tumors under the filtering conditions of adjusted *p*‐value (P.adj) < 0.05 and log fold change (logFC) ≥ 0.8 (Figure 3D). To further elucidate the molecular features of functional ACAs, a functional enrichment analysis was conducted on these differentially expressed genes (Figure 3E; Figure S5D–F and Table S6, Supporting Information). GO enrichment analysis of biological processes revealed that upregulated genes were predominantly enriched in pathways related to glucocorticoid metabolism and hormone biosynthetic processes (Figure 3E).

The CLU gene, which encodes clusterin (also known as apolipoprotein J), is predominantly secreted by the brain and various endocrine organs, including the adrenal glands. Clusterin has been implicated in recovery processes following brain and renal injuries.^[^ ^26^ ^]^ Additionally, it has been identified as a secreted marker associated with an HIF‐independent pathway regulated by the von Hippel‐Lindau (VHL) tumor suppressor protein.^[^ ^27^ ^]^ Notably, CLU emerged as one of the most prominently differentially expressed genes in both non‐functional and functional ACA groups (Figure S5A,B, Supporting Information).To further substantiate this observation, the correlation between CLU expression and two established markers, CYP11B2 and HSD3B2, was examined in ACAs (Figure 3F,G). Significant positive correlations were identified, with correlation coefficients of 0.46 and 0.77, respectively. In this study, elevated expression levels of CLU were detected in both non‐functional and functional ACA samples (Figure 3H). Immunohistochemical (IHC) analysis confirmed the co‐localization of CLU, CYP11B2, and HSD3B2 in ACAs, with CLU displaying a broader expression pattern compared to the other two markers (Figure 3I). Collectively, the ACAs analysis with varying hormonal abnormalities at the single cell level identified potential pathological diagnostic markers.

### 2.4 Transcriptional Regulation in Adrenocortical Tumor Cells

**Figure 4:**
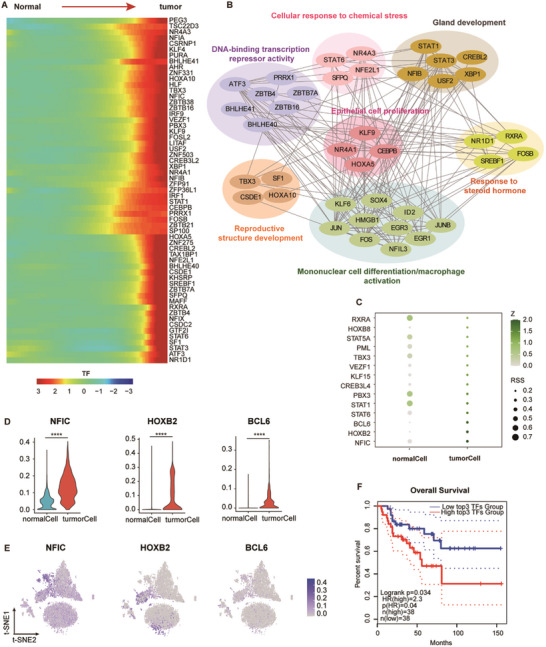
Transcription‐factor regulatory networks and key activators of adrenocortical tumor cells. A) Heatmap showing TF expression along the pseudotime trajectory from normal to tumor cells. Normalized expression values are presented, with color gradients indicating expression levels (red: high; blue: low). B) Gene‐gene interaction network of TFs identified in A). GO functional annotations highlight pathways involved in DNA‐binding transcription repressor activity, epithelial cell proliferation, reproductive structure development, cellular response to chemical stress, response to steroid hormones, mononuclear cell differentiation/macrophage activation, and gland development. Edges represent interaction confidence derived from STRING database analysis. C) Dot plot showing 14 Transcription Start Site (TSS) TF motifs enriched in tumor cells, identified through SCENIC analysis. Regulatory strength is represented by RSS values, and dot size indicates relative significance. D,E) Violin plots D) and t‐SNE projections E) showing expression levels of NFIC, HOXB2, and BCL6 in tumor cells compared to normal cells. **** *p ≤ 0.0001*. F) Kaplan‐Meier survival curves showing the relationship between the expression levels of highly expressed top3 TFs in tumor cells and overall survival in adrenocortical carcinoma (ACC) patients. High‐expression (n = 38) and low‐expression (n = 38) groups were stratified using the median value as a cutoff. Hazard ratios (HR) and log‐rank test *p*‐values are shown.

As previously demonstrated, hyperfunctional ACAs produce excessive corticosteroids due to dysregulated expression of steroidogenic enzymes. Although no genetic mutations have been identified in steroidogenic enzyme genes, dysregulated expression at the transcriptional level might be critical. To investigate this, TF expression differences between normal and tumor cells were analyzed (**Figure** **4**A). Interaction network analysis of upregulated TFs in tumor cells revealed that upregulated TFs are associated with pathways related to DNA‐binding transcription repressor activity, epithelial cell proliferation, reproductive structure development, cellular response to chemical stress, mononuclear cell differentiation/macrophage activation, response to steroid hormones, and gland development. These results highlight the crucial roles of these TFs in tumor progression (Figure 4B). Among these, SF1 was notably upregulated in tumor cells. SF1 regulates steroidogenic enzymes, including STAR, CYP11A1, and MC2R, and is associated with adrenal cortical tumors.^[^ ^28^, ^29^, ^30^ ^]^ To build on these findings, the expression profiles of 125 downregulated genes and 83 upregulated genes following the knockdown of SF‐1 from public data were analyzed (GSE43035) (Figure S6A,B, Supporting Information). The results revealed a significant enrichment of the downregulated genes in ACAs. Previous studies have indicated that genes negatively regulated by SF‐1 (which is activated by SF‐1) are predominantly involved in lipid and steroid metabolism.^[^ ^31^ ^]^ Subsequently, IHC staining was employed to assess SF1 expression and its distribution pattern (Figure S6B, Supporting Information). Elevated SF1 expression was detected in non‐functional adenomas and Conn's syndrome samples, whereas its expression was nearly absent in PCC tumors (Figure S6C, Supporting Information). Collectively, these findings suggest that SF1 serves as a highly sensitive and specific immunohistochemical marker for identifying tumors of adrenocortical origin.

Furthermore, TFs involved in the transition of cortical cells from a normal to a tumor phenotype were identified (Figure S6D, Supporting Information). These TFs were linked to pathways such as epithelial cell proliferation, neural development, and hormone biosynthetic processes (Figure S6E, Supporting Information). The results suggest that TF expression undergoes substantial alterations during the progression from adrenocortical organogenesis to tumorigenesis. To explore the prognostic significance of these TFs, single‐cell regulatory network inference and clustering (SCENIC) was employed,^[^ ^32^ ^]^ leading to the identification of 14 TFs that were significantly enriched in both the tumor state and regulon analysis of tumor cells (Figure 4C; Figure S6F, Supporting Information). Among these, the top three TFs—NFIC, HOXB2, and BCL6—demonstrated notably high expression in tumor cells (Figure 4D). To assess the prognostic relevance of these TFs, a survival analysis was performed using ACC samples via the GEPIA2 platform (Figure 4F). The results indicated a strong correlation between the top 3 tumor‐specifically enriched TFs and poor clinical outcomes in ACC patients. These findings align with previous research, highlighting the critical role of transcription factors in driving tumorigenesis and the progression of adrenocortical tumors.

### 2.5 Association of MYCN Expression with Prognosis and Metastatic Risk in PCC

**Figure 5:**
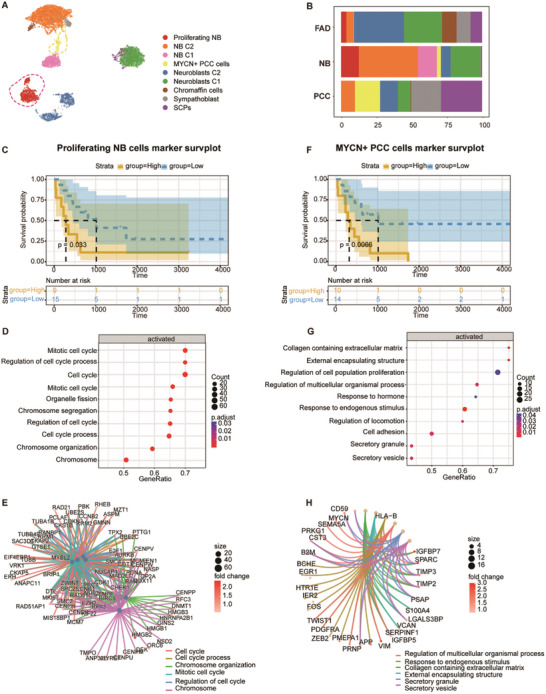
Characterization and Functional Identification of Mueller Cells Associated with Poor Survival. A) UMAP plot depicting detailed annotation of Mueller cell subpopulations based on a Mueller cell‐specific joint embedding across multiple adrenal tumor datasets. Cell types include SCPs, sympathoblasts, chromaffin cells, neuroblasts (C1 and C2), MYCN+ PCC cells, NB (neuroblastoma) cells (C1 and C2), and proliferating NB cells. B) Bar plot showing the proportional distribution of preliminary cell types within FAD, NB, and PCC, colored according to cell type annotations in A). C) Kaplan‐Meier curves for overall survival of 498 cases in the NB cohort (GSE10927) of the markers in proliferating NB cells. D) Dot plots showing the top enriched GO pathways for proliferating NB cells using Gene Set Enrichment Analysis (GSEA). GeneRatio indicates the proportion of genes involved in each pathway. Adjusted *p*‐values (p.adjust) are color‐coded, with p.adjust ≤0.05 considered significant. E) Gene‐gene interaction networks illustrating key genes involved in the enriched pathways for proliferating NB cells. Nodes represent genes. Node color is proportional to fold change values, and the pathways are labeled by color. F) Kaplan‐Meier curves for overall survival of 498 cases in the NB cohort (GSE10927) of the markers in MYCN+ PCC cells. G) Dot plots showing the top enriched GO pathways for MYCN+ PCC cells using GSEA. H) Gene‐gene interaction networks illustrating key genes involved in the enriched pathways for MYCN+ PCC cells.

The PCC and NB are tumors, both arising from the adrenal medulla cell lineage, and exhibit shared certain genetic characteristics.^[^ ^33^ ^]^ To gain deeper insight into the malignant properties of these tumors, a secondary clustering analysis was conducted on medullary cells (**Figure** **5**A). This analysis identified a cluster of NB cells characterized by enhanced cell cycle features, characterized by elevated expression of genes associated with cell cycle regulation, including *TOP2A*, *CCNB1*, and *MKI67* ^[^ ^9^, ^34^ ^]^ (Figure S7A, Supporting Information). These cells were consequently classified as proliferating NB cells. Consistent with previous research, patients with a high proportion of proliferating neuroblastoma‐like tumor cells were associated with poorer patient outcomes (Figure 5C).^[^ ^34^ ^]^ Additionally, pathway enrichment analysis suggested that the functional abnormalities of proliferating NB cells may be associated with the activation of pathways and genes related to the cell cycle (Figure 5D,E).

Notably, a distinct cell population was identified that exhibited elevated *MYCN* expression levels within PCC samples (Figure 5B; Figure S7A, Supporting Information). MYCN amplification has been previously demonstrated to correlate with tumor progression and increased malignant potential in PCC.^[^ ^35^ ^]^ Multivariate Cox regression analysis of overall survival demonstrated that the gene expression signatures of this specific cell type could predict patient prognosis based on MYCN amplification status (Figure 5F). Furthermore, MYCN‐amplified PCC was associated with a young age at onset, larger tumor size, and a higher risk of metastasis.^[^ ^36^, ^37^ ^]^ To corroborate these observations, single‐cell data from four previously published PCC cases were integrated.^[^ ^14^ ^]^ This analysis revealed that two PCC cases from the current study displayed a stronger alignment with the metabolic PCC subtype, known for its higher metastatic potential (Figure S7B,C, Supporting Information). Compared to kinase‐type PCC, which has a lower likelihood of metastasis, the two PCC samples from this study, along with three kinase‐type metabolic PCC cases, exhibited significantly higher scores for MYCN cell population markers (Figure S7D,E, Supporting Information). Additionally, pathway enrichment analysis indicated that the functional abnormalities of MYCN+ PCC cells might be associated with the activation of pathways related to endogenous stimulus, collagen‐containing extracellular matrix, and external encapsulating structure (Figure 5G,H). These findings suggest that MYCN expression level holds significant prognostic value and may contribute to the development of targeted clinical management strategies for PCC patients.

### 2.6 Immune Microenvironment and Signaling Pathway Alterations in Benign and Malignant Adrenal Tumors

**Figure 6:**
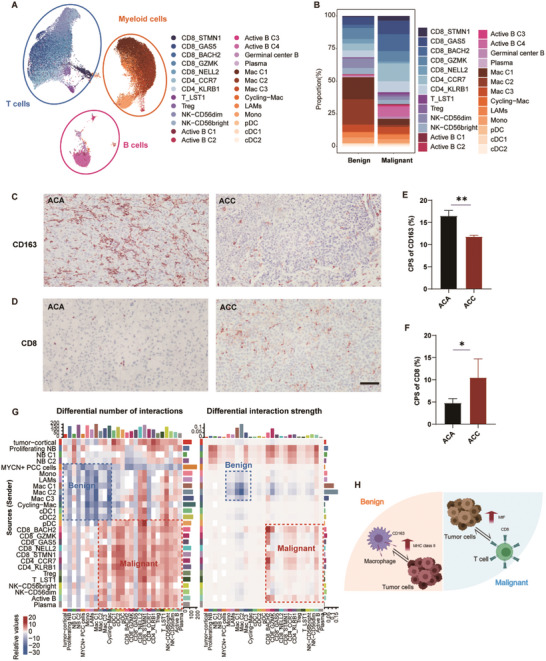
Tumor‐immune cell interactions and ligand‐receptor signaling in benign and malignant tumors. A) UMAP plot showing detailed annotation of immune cell subpopulations based on immune cell‐specific joint embedding. The identified clusters include CD8+ T cells, CD4+ T cells, B cells, NK cells, macrophages (Mac C1‐C3), monocytes (Mono), plasma cells, and dendritic cells (cDC1 and cDC2), among others. B) Bar plot illustrating the proportional composition of immune cell subtypes in benign and malignant tumors. C,D) Immunohistochemistry images showing the expression of CD163 C) and CD8 D) in ACAs and adrenal cortical carcinoma (ACC). Scale bar, 100 µm. E,F) Immunohistochemistry results showing the expression levels of CD163 E) and CD8 F) in ACAs and ACC. CPS: Combined positive scores of immune marker. * *p* ≤ 0.05, ** *p* ≤ 0.01. G) Heatmaps representing the differential number of interactions (left) and interaction strengths (right) between immune cell clusters and tumor cells. Blue indicates higher communication in benign tumors, while red represents stronger communication in malignant tumors. Data are derived from CellChat analysis and normalized interaction scores. H) Schematic diagram illustrating the shifts in interactions of tumor‐infiltrating immune cell subsets in benign and malignant tumors.

Several studies have consistently demonstrated the critical role of immune cell composition, quantity, and functional status in the progression and prognosis of adrenal tumors.^[^ ^38^, ^39^ ^]^ Our previous inferCNV analysis revealed increased inferCNV scores in immune cells, particularly in T cells and macrophages (Figure S3A, Supporting Information), suggesting potential abnormalities in immune function and response. To gain a comprehensive understanding of the immune microenvironment's differences between benign and malignant adrenal tumors, we first evaluated the immune cell populations. This analysis revealed a diverse range of immune subtypes, encompassing myeloid, B cell, and T cell lineages, with a total of 26 distinct clusters identified (**Figure** **6**A). In benign tumors, a higher proportion of myeloid immune cells, such as macrophages, was observed, while malignant tumors demonstrated a greater proportion of CD8+ T cells (Figure 6B). Immunohistochemical staining of macrophage marker (CD163) and CD8+ T cell marker (CD8) in benign ACAs and malignant adrenal cortical carcinomas (ACC) revealed that CD163 expression was significantly higher in benign tumors, while CD8 expression was markedly elevated in malignant tumors (Figure 6C–F), aligning with the previous findings.

To further investigate the signaling pathways influencing immune regulation in benign and malignant adrenal tumors, CellChat was employed to explore the principal signaling inputs and outputs between immune cells and tumor cells^[^ ^40^ ^]^ (Figure 6G; Figure S8A,B, Supporting Information). In malignant tumors, significant interactions were detected between T cell subtypes (CD4+, CD8+, and NK cells) (Figure 6G). In contrast, benign tumors predominantly exhibited enhanced interactions among myeloid cell subtypes, particularly involving macrophages (Figure 6G). Macrophage migration inhibitory factor (MIF) signaling, which has been associated with decreased tumor survival and immune modulation,^[^ ^41^, ^42^ ^]^ was identified as originating primarily from NB cells, with B lymphocytes acting as the main recipients in malignant tumors (Figure S8C, Supporting Information). In contrast, benign adrenal tumors exhibited increased signaling through the major histocompatibility complex class II (MHC II) pathway, where macrophages acted as both signal emitters and recipients, while tumor cells lacked detectable MHC II signaling activity (Figure S8A,D, Supporting Information). Previous studies have indicated that MHC II deficiency can impair the ability of tumor cells to be recognized and to elicit an immune response, thereby facilitating immune evasion.^[^ ^43^, ^44^ ^]^ These findings are of critical significance for understanding immune responses and TME in benign and malignant adrenal tumors.

## 3 Discussion

The early diagnosis of adrenal tumors presents significant challenges, as they are frequently discovered incidentally during unrelated medical investigations. Functional tumors, which produce hormones and exhibit diverse endocrine symptoms, further complicate diagnostic efforts.^[^ ^1^ ^]^ This study draws a single‐cell atlas to uncover the cellular heterogeneity and molecular characteristics of various adrenal incidentaloma (AI) subtypes (Figure 1). Our findings demonstrate substantial alterations in the secretory functions of cortical tumor cells compared to normal adrenal cortical cells (Figure 2). The study highlights the progressive changes in gene expression and biological processes during the transformation from normal to tumorigenic cortical cells.

Single‐cell analysis revealed significant heterogeneity in the distribution of cortical cells within the specimens (Figure 3). Differential gene expression analysis identified a notable upregulation of steroidogenic enzyme genes in ACAs. Additionally, a unique cortical cell subtype was identified in ACAs, characterized by elevated expression of the CLU gene (Figure 3). Accurate preoperative diagnosis is crucial for determining surgical strategies and predicting postoperative outcomes; however, challenges in obtaining representative tissue samples may limit diagnostic precision. Clusterin, the protein encoded by CLU, shows significant potential as a diagnostic biomarker for ACAs. Its detection via non‐invasive methods, such as blood or urine sampling, offers significant advantages in terms of patient convenience and safety while enhancing the feasibility of screening and monitoring patients with aldosterone overproduction.^[^ ^45^, ^46^ ^]^ Moreover, Clusterin can complement established biomarkers, such as CYP11B2 and HSD3B2, to improve diagnostic accuracy and specificity. However, further research is needed to validate its utility in multiparametric diagnostic panels.

MYCN encodes a core transcription factor essential for embryonic development and the normal physiological processes of the nervous system, playing a critical role in regulating the proliferation and differentiation of neuroblasts.^[^ ^47^ ^]^ In neuroblastoma (NB) patients, MYCN amplification is typically associated with increased tumor aggressiveness and malignancy.^[^ ^48^ ^]^ A comprehensive analysis of medullary cells identified a population of PCC cells with elevated MYCN (N‐Myc) expression (Figure 5). Previous studies have classified PCC into two subtypes: the highly metastatic metabolically driven PCC and the less aggressive kinase‐driven PCC.^[^ ^14^ ^]^ Comparative analysis of the MYCN+ PCC cell population revealed that elevated MYCN expression correlates with the highly metastatic metabolic subtype. These findings suggest that MYCN may hold potential prognostic value in PCC, offering a promising avenue for further investigation and therapeutic intervention.

The differential diagnosis between benign and malignant adrenal tumors remains a significant clinical challenge, as misdiagnosis can profoundly impact treatment strategies. The complexity of the tumor microenvironment (TME) further complicates distinguishing between these tumor types. To minimize the potential influence of immune activation, commonly observed in inflammatory tumor microenvironments, fetal adrenal tissue was selected as the reference for inferCNV analysis. This choice enabled more precise differentiation between tumor‐associated transcriptional programs and artifacts of immune activation (Figure 6; Figure S3, Supporting Information). Immunohistochemical validation highlighted distinct differences in the immune microenvironment between benign and malignant adrenal tumors. Myeloid markers (e.g., CD163) were predominantly expressed in benign adrenal cortical adenomas, whereas lymphoid markers (e.g., CD8) were elevated in malignant tumors. These observations align with findings from single‐cell transcriptomic studies, underscoring the relationship between immune cell composition and tumor progression.

This study has certain limitations, such as the use of fetal adrenal tissue as a reference, due to the current lack of publicly available single‐cell datasets of normal adult adrenal tissues. While the developmental state of fetal adrenal tissue may not fully correspond to that of adult adrenal glands, it still provides a valuable foundation for investigating developmental transcriptional programs and identifying malignant cell states, particularly in adrenal medulla‐derived tumors such as pheochromocytomas and neuroblastomas. Future studies incorporating single‐cell data from normal adult adrenal tissues will be instrumental for further validating these findings and refining the conclusions. The pathogenesis and progression of adrenal tumors represent a complex process influenced by multiple factors that extend beyond transcriptional regulation and the tumor microenvironment. Nevertheless, these findings carry significant clinical implications, offering a foundation for future studies aimed at improving patient outcomes and advancing the management of adrenal incidentalomas.

## 4 Experimental Section

### Single Cell Preparation and Flow Cytometry

The study involved a cohort of 11 patients (7 females, 4 males; aged 29–67 years, mean age 47.5 years) diagnosed with adrenal incidentalomas. Detailed demographic and clinical data (Table S1, Supporting Information) provided a comprehensive overview. Eligibility was limited to patients undergoing surgical resection for adrenal incidentalomas; those with previously treated or recurrent tumors were excluded to minimize confounding variables.

Tumor samples were processed under cold conditions using phosphate‐buffered saline (PBS). After mechanical disaggregation into ≈1 mm^3^ fragments, tissues were enzymatically digested with collagenase IV and DNase I in a DMEM/F12 medium for 30 min at 37 °C. Cellular debris was removed using a 70‐µm filter, and cells were washed with PBS containing 1% bovine serum albumin (BSA) and 2 mm ethylenediaminetetraacetic acid (EDTA). Red blood cells were eliminated via density gradient centrifugation, and viability was assessed using a Countess instrument. Live and non‐erythroid cells were sorted using Calcein AM and DARQ5 markers on a BD FACS Aria III.

### Single Cell Library Preparation and RNA Sequencing

Single‐cell libraries were constructed using the 10x Genomics Chromium platform or SeekOne DD kit. Cell viability and concentration were measured with an automated fluorescence counter, and samples were adjusted to 1000 cells µL^−1^. Libraries were sequenced using paired‐end, 150 bp reads on an Illumina NovaSeq 6000 platform.

### Preprocessing and Quality Control

A total of 87 163 cells from 11 patients included in this study were sequenced. Low‐quality cells were excluded based on criteria of fewer than 300 detected genes and/or mitochondrial gene content exceeding 20%. As a result, 62784 high‐quality cells were retained. Sequencing data were processed with the GRCh38 reference genome and filtered using criteria including doublet detection via the DoubletFinder (v.2.0.3) R package. Final datasets were normalized and scaled for downstream analyses using Seurat (v.4.4.0).^[^ ^49^ ^]^ Similarly, the raw data from the public dataset GSE137804 were aligned and quality‐controlled under the same reference genome and filtering conditions.

### Integration and Dimensionality Reduction

Single‐cell datasets were integrated using Seurat's canonical correlation analysis (CCA) pipeline. Batch effects were mitigated, and dimensionality reduction was performed using Uniform Manifold Approximation and Projection (UMAP) and t‐distributed Stochastic Neighbor Embedding (tSNE). Cell types were annotated based on established marker genes (see Table S3, Supporting Information).

### Differential Gene Expression and Pathway Analysis

Differentially expressed genes (DEGs) were identified using the Wilcoxon Rank‐Sum test. DEGs were filtered based on statistical thresholds (e.g., Log2FoldChange ≥ 0.8, adjusted *p* < 0.05). Gene regulatory networks were analyzed using pySCENIC (v.0.12.1),^[^ ^50^ ^]^ and pathway annotations were performed using clusterProfiler (v.4.6.2).^[^ ^51^ ^]^ Results were visualized through STRING (v.12.0.0)^[^ ^52^ ^]^ and Cytoscape (v.3.8.2).^[^ ^53^ ^]^

### Inferred CNV Analysis from scRNA‐seq

To identify malignant cells, CNVs were inferred at the single‐cell level using smoothed gene expression profiles across chromosomal regions. CNV analysis was conducted with the InferCNV R package (v.1.14.2)^[^ ^54^ ^]^ under the parameters cutoff = 0.1, denoise = TRUE, and HMM = TRUE. Normal tissue cells served as reference points. To distinguish the chromosomal gene expression patterns of cancer cells from those of non‐cancer cells, normal fetal adrenal tissue (GSE137804) was utilized for comparison. Z‐scores were calculated by normalizing gene expression data against the average profiles of reference tissues. Genes were then ordered based on chromosomal position and starting coordinates.

### Survival Analysis

Prognostic performance of individual genes and cell clusters was evaluated using patient datasets (n = 498, GEO: GSE49710; n = 65, GEO: GSE10927). For specific genes, survival data were analyzed through the R2 Genomics platform (http://r2.amc.nl).^[^ ^55^ ^]^ A threshold for significant survival differences was determined using the platform's scanning function, which informed the Kaplan–Meier curve generation. Survival analysis at the cluster level was performed using the Cox proportional hazards regression model implemented in the R survival package. Kaplan–Meier plots were visualized with the survminer package.

### Immunohistochemistry and Fluorescence In Situ Hybridization

Paraffin‐embedded tissue sections underwent antigen retrieval, immunostaining, and counterstaining using standard protocols.^[^ ^7^ ^]^ Fluorescence in situ hybridization employed custom probes targeting HSD3B2, CYP11B2, and CLU, and imaging was conducted using a Leica Dmi8 microscope.

### Statistical Analysis

Statistical analyses were performed using R software (v.4.3.0) for scRNA‐seq datasets and GraphPad Prism (v.9.3) for imaging data. Data were pre‐processed by normalizing to 10 000 UMIs per cell and log‐transforming using the Seurat Normalize Data function. Outliers were evaluated based on quality control thresholds, and low‐quality cells were excluded from the analysis. Data are presented as mean ± SD for continuous variables. Sample sizes for each statistical analysis were specified in the respective figure legends. Statistical tests were chosen based on the data type and analysis objective, with linear models applied to continuous data and *t*‐tests or Wilcoxon rank‐sum tests used for pairwise comparisons. For untargeted analyses, such as differential gene expression, transcription factor activity, or pathway enrichment, false discovery rate (FDR) adjustments were applied to p‐values. Assumptions for the statistical tests, such as normality and homogeneity of variance, were verified before analysis. Specific details on the tests and methods used are provided in the figure legends.

### Ethics Approval and Consent to Participate

All study participants provided written informed consent and study protocols were approved by the ethical review community of  Shanghai General Hospital affiliated to Shanghai Jiao Tong University School of Medicine (No. 2024SQ538). All the procedures have been performed as per the ethical guidelines laid down by the Declaration of Helsinki. Informed consent was obtained from all subjects.

### Availability of Data

All of data generated or analyzed in this study are included in the manuscript. The raw sequencing data of this paper are archived on the Genome Sequence Archive for Human of National Genomics Data Center: https://ngdc.cncb.ac.cn/gsa‐human/ with accession number: HRA005846. The code information in this study can be provided upon reasonable request by contacting the corresponding authors.

## Conflict of Interest

The authors declare no conflict of interest.

## Author Contributions

M.W., G.Z., X.H., and F.T. contributed equally to the work. C.Z. conceived and designed the study; M.W., G.Z., and F.T. conducted the scRNA‐seq analysis; X.H., L.Y., and Y.Q. performed experiments; Z.Z., K.G., S.C., X.F., and Y.S. performed the in‐silico analysis; T.L., L.L., D.C., L.L., and F.L. collected the samples and patient information; M.W. and G.Z. wrote the manuscript and performed data interpretation, with input from C.Z., B.L., and R.Z.; C.Z. and R.Z. made the final revision. All authors agreed on the final version of the manuscript.

## Supporting information



Supporting Information

Supplemental Table 1

Supplemental Table 2

Supplemental Table 3

Supplemental Table 4

Supplemental Table 5

Supplemental Table 6

Supplemental Table 7

Supplemental Table 8

Supplemental Table 9

## Data Availability

The data that support the findings of this study are available from the corresponding author upon reasonable request.
