# Supplementary material for: Single‐Cell Atlas Reveals Tumorigenic Profiles and Immune Dynamics of Adrenal Incidentalomas
Source: Adv Sci (Weinh). 2025 Apr 7;12(22):2413493. doi: 10.1002/advs.202413493 (PMC12165089; doi:10.1002/advs.202413493)
Supplement: Supplementary file 1 — Supporting Information [file ADVS-12-2413493-s009.docx]

## Supplementary Figures and Tables


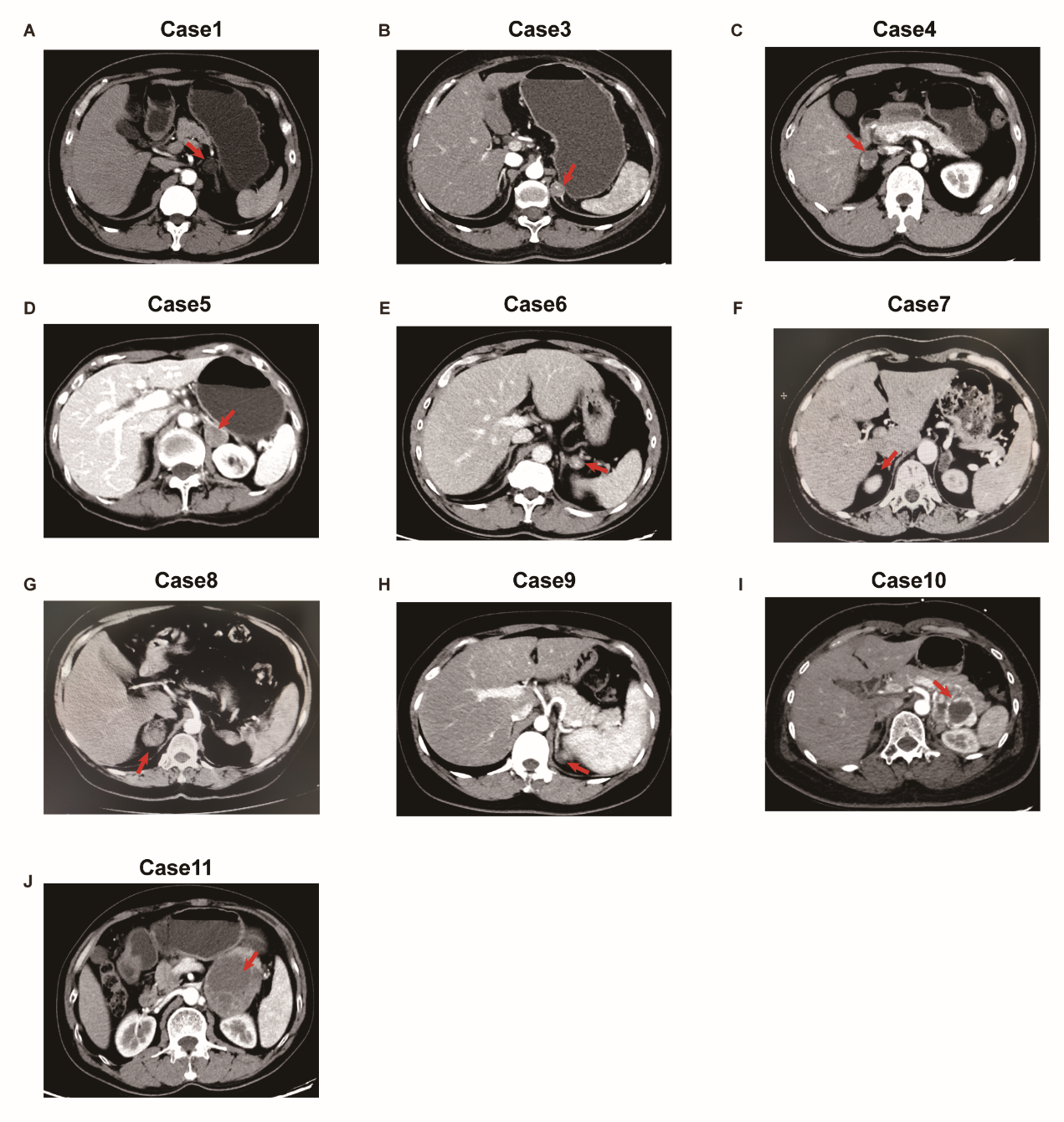


### Figure S1. Diagnosis of Adrenal Incidentaloma by Contrasted CT

(A-J) Contrasted CT scanning for 11 AI cases. The red arrow indicates the location of the tumor.


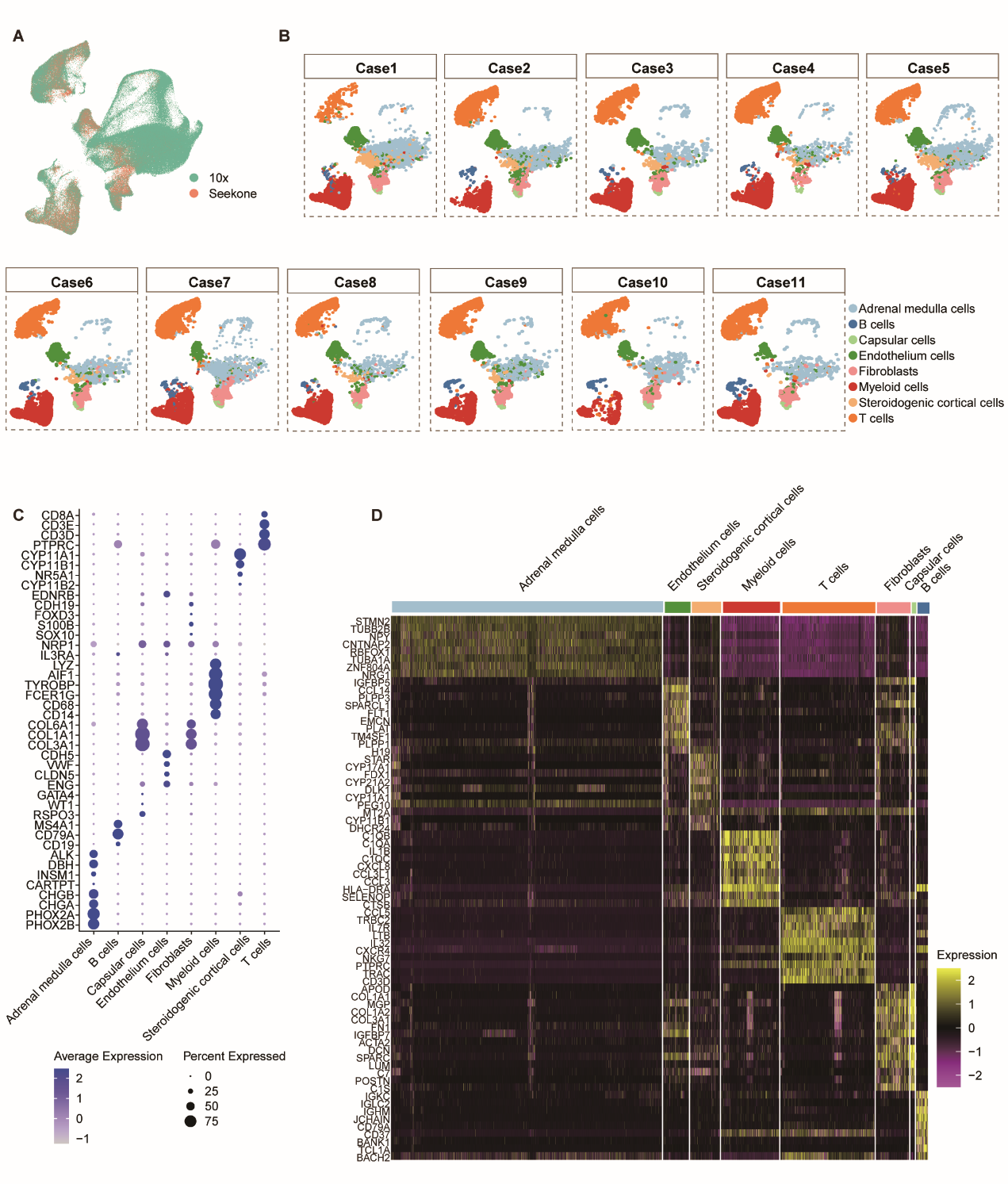


### Figure S2. Cellular Diversity within the 11 AI Samples

(A) UMAP visualization showing an integrated mapping of two sequencing platforms (Green: 10x Genomics; Orange: Seekone).

(B) UMAP plots showing the distribution of identified cell types in adrenocortical incidentaloma (AI) samples (n = 11). Each color represents a distinct cell type, consistent across all figures.

(C) Dot plot representing the expression of known marker genes in eight cell types. The color represents the scaled average expression of marker genes in each cell type, and the size indicates the proportion of cells expressing marker genes.

(D) Heatmap indicates the scaled expression patterns of the top 5 differentially expressed genes in each cell type. Relative expression levels are color-coded (yellow = high, purple = low).


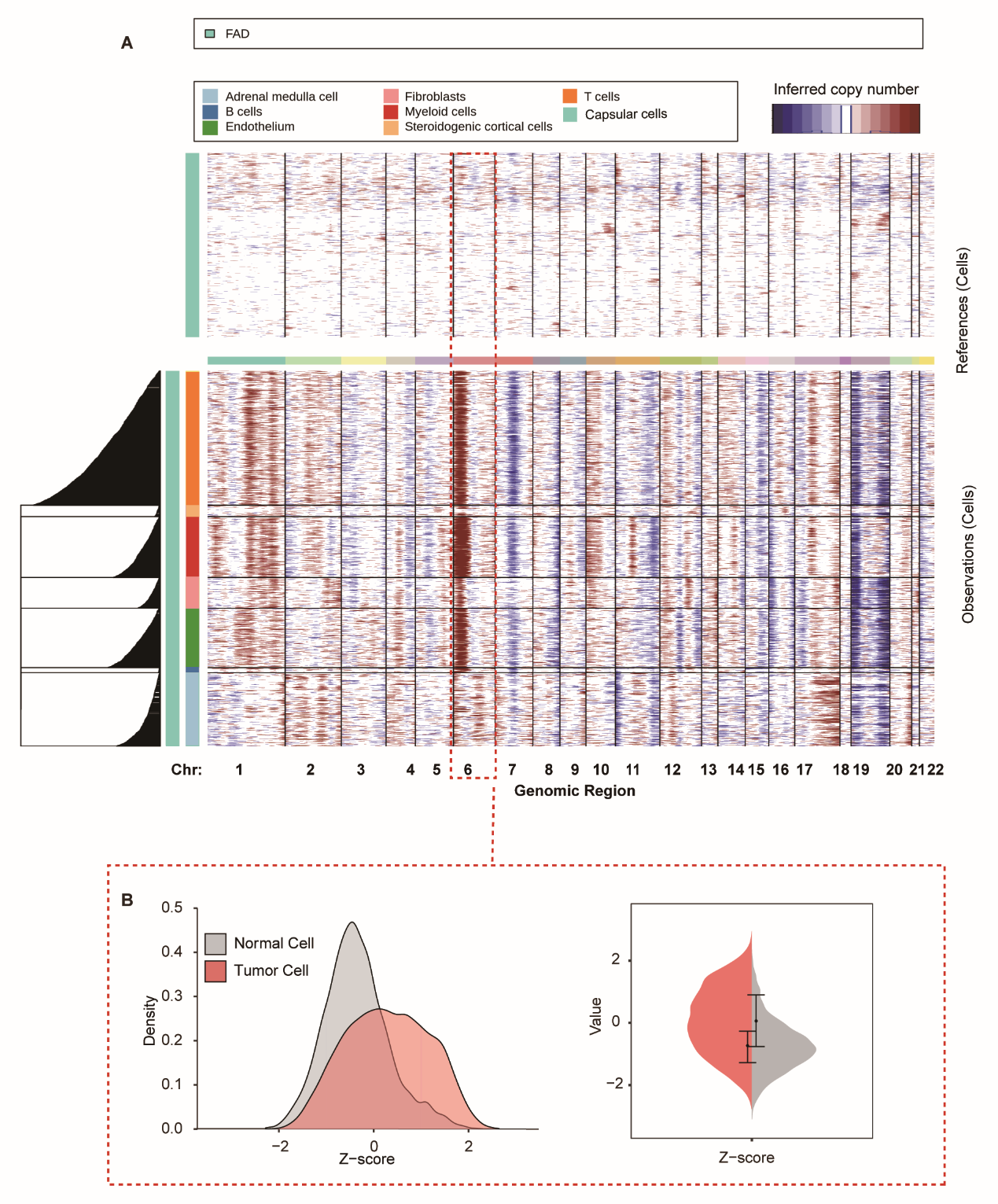


### Figure S3. Separation of tumor and non-tumor cells

(A) Inferred copy number based on scRNA-seq data. Adrenal cells from FAD are a normal reference for CNV (Copy Number Variation) inference of malignant cells. The red color represents a high CNV level, and the blue represents a low CNV level.

(B) Left: The density of normal and tumor cells for the Chr6 region. Right: Vlnplot visualization 100-gene rolling average expression for each cell. Red: tumor cells, Gray: normal cells.


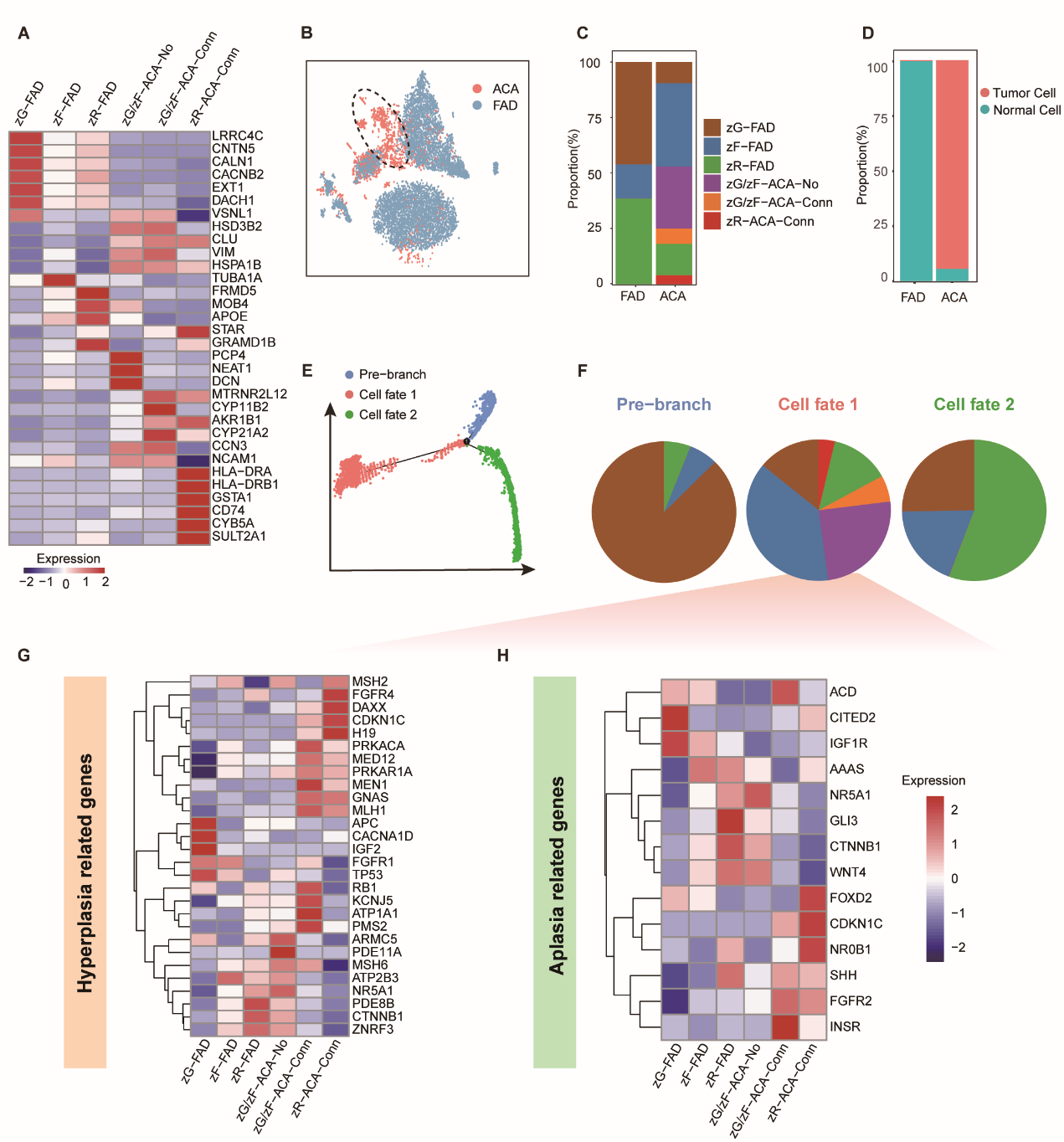


### Figure S4. Characterization of Adrenocotical Cell Lineages

(A) Heatmap shows the normalized mean expression of marker genes (rows) across different adrenocortical cell subclusters, categorized by pathology and cortex layers.

(B) t-SNE visualization showing adrenocortical cell joint embedding. Circles highlight the main distribution of AI samples in adrenocortical clusters.

(C) Frequency distribution of adrenocortical subtypes among FAD and ACA samples.

(D) Frequency distribution of normal and tumor cells among FAD and ACA samples.
(E) Trajectory plots illustrating the cellular states across three branches of steroidogenic cortical cells, indicating pseudotime progression from pre-branch to two distinct cell fate paths.

(F) Pie plots displaying the distribution of adrenocortical cell subpopulations in pre-branch, cell fate 1, and cell fate 2, reflecting differentiation trajectories.

(G-H) The heatmap shows the normalized mean expression of hyperplasia-related (G) and aplasia-related genes (H) across different adrenocortical cell subclusters.


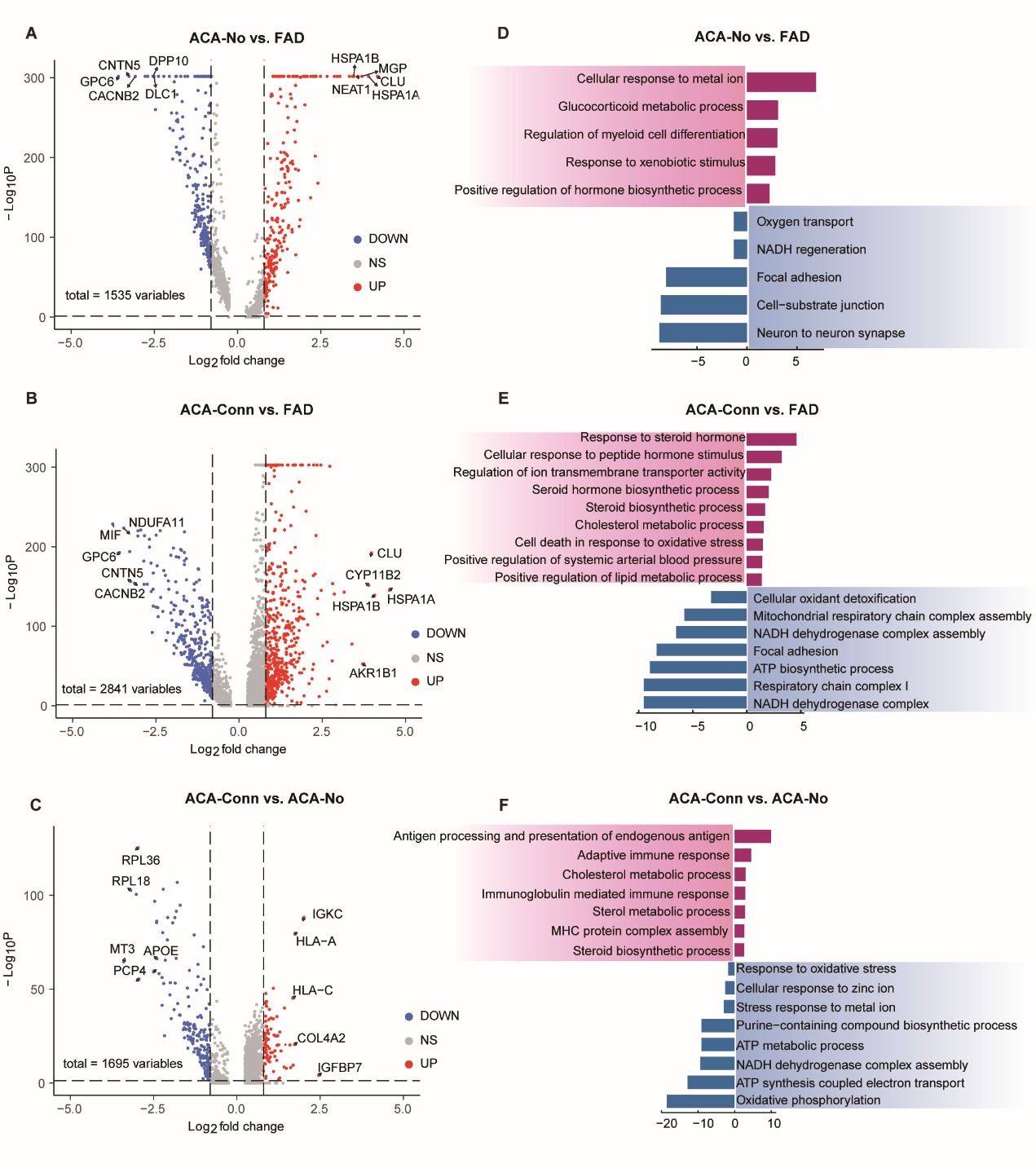


### Figure S5. Differential Gene Expression Analysis Reveals Functional Changes in ACAs.

(A-C) Volcano plots display changes in gene expression in adrenocortical cells by comparing ACA-No vs. FAD (A), ACA-Conn vs. FAD (B), and ACA-Conn vs. ACA-No (C). FAD: fetal adrenal samples; ACA-No: No functioning adenoma; ACA-Conn: Conn's syndrome. The x-axis represents the natural logarithm of fold-changes (Fold-Changes, FC), while the y-axis represents the negative logarithm of the adjusted p-values (base 10). Dashed vertical and horizontal lines highlight the filtering thresholds, defined as p.adjust ≤ 0.05 and logFC > 0.8. Red and blue dots represent top 5 significantly upregulated and downregulated genes, respectively.

(D-F) Functional enrichment analysis highlights the functional changes in upregulated or downregulated genes in adrenocortical cells based on comparisons between ACA-No vs. FAD (D), ACA-Conn vs. FAD (E), and ACA-Conn vs. ACA-No (F). The most significantly altered terms in the GO biological processes are shown. The x-axis represents the negative logarithm of the adjusted p-values (base 10).


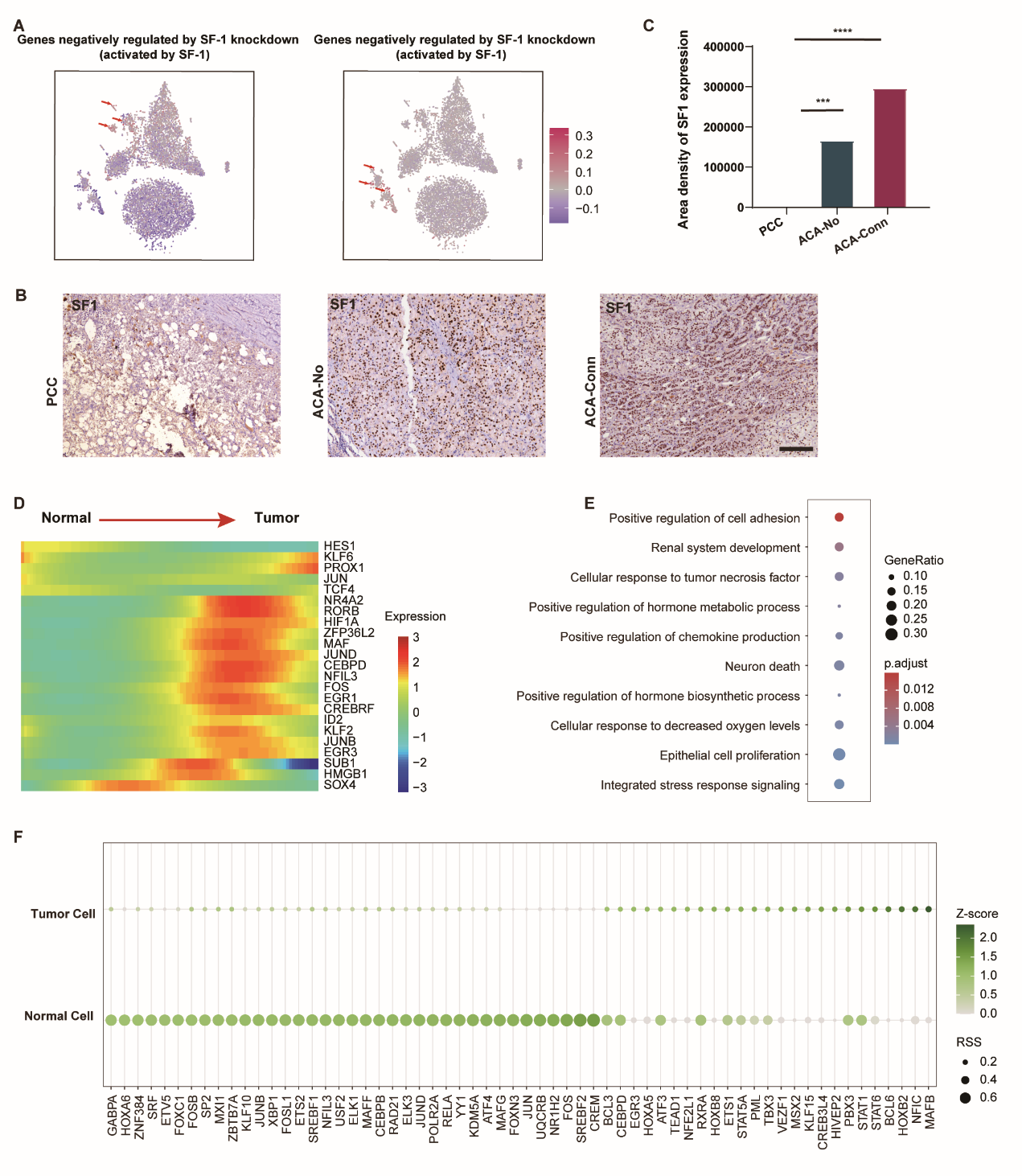


### Figure S6. SF1 and SCENIC Analysis in Adrenocortical Tumors

(A) t-SNE plot showing the average gene expression score of the SF1 transcription factor dosage regulated in adrenocortical cancer cells. The red arrows indicate the cells with high gene expression.

(B) IHC images representing SF1 staining in various adenoma tumor samples. PCC: Pheochromocytoma; ACA-No: non-functional adenoma; ACA-Conn: Conn's syndrome. Scale bar, 100 μm.

(C) Quantitative analysis of SF1 expression in non-functional adenoma and Conn's syndrome patients based on IHC results.

(D) Heatmap showing the distribution of representative TFs across pseudotime trajectory states. The arrow indicates the direction of pseudotime.

(E) Top enriched pathways identified during the transition from normal tissue to tumor tissue, determined through GO analysis.

(F) Tumor and normal cell specific regulatory analysis conducted using SCENIC. The heatmap is colored according to the regulon specificity score (RSS) showing the specificity of the activated TF.


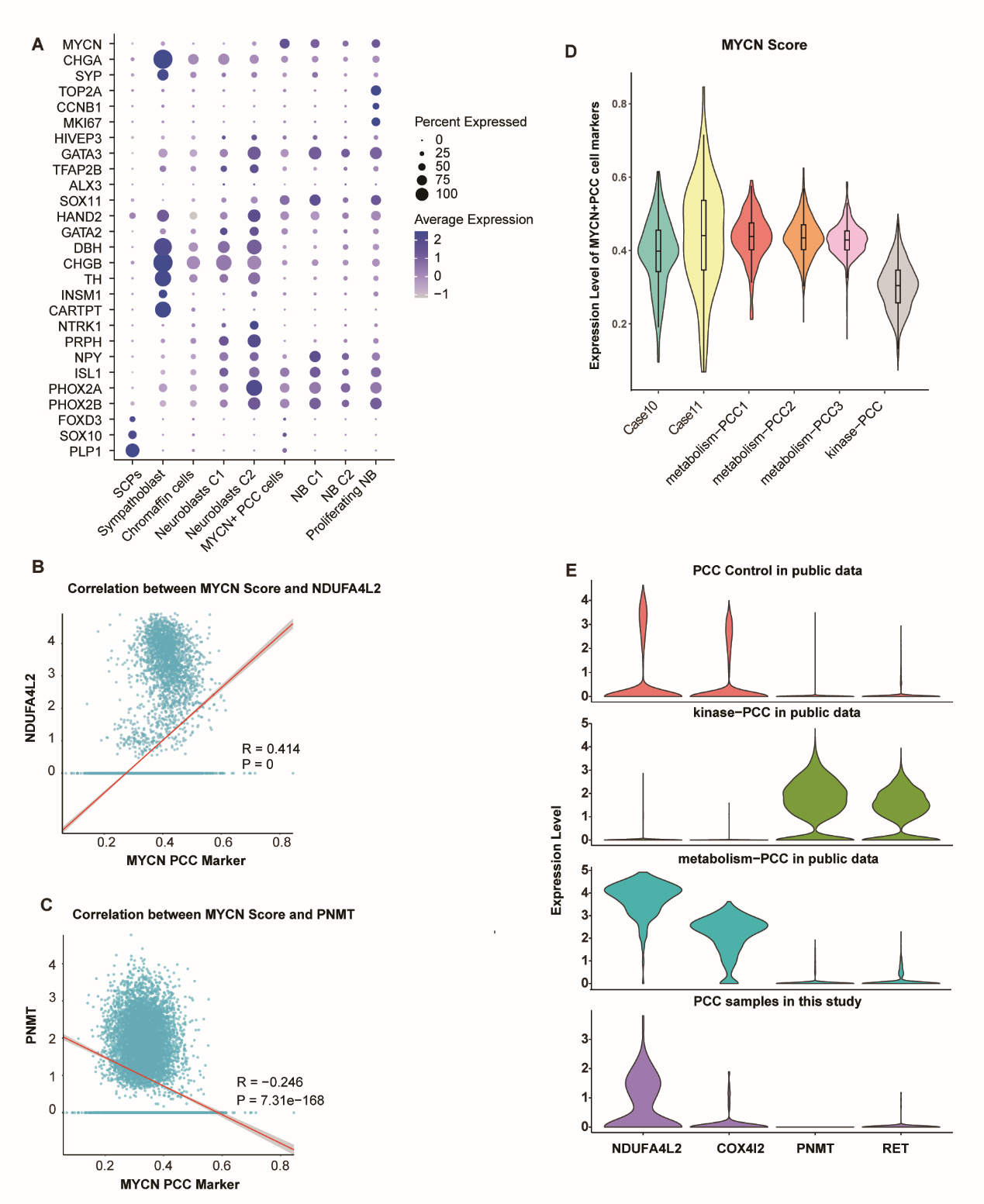


### Figure S7. Characterization of Medullary Cell Subtypes and Features of MYCN+ PCC cells

(A) Dot plot showing the expression levels of key differentially expressed marker genes across various medullary cell subtypes. Dot size corresponds to the proportion of cells expressing each gene, while color intensity reflects the average expression level.

(B) Scatter plots showing the correlation between MYCN+ PCC cells markers’ score and the expression levels of (B) *NDUFA4L2* and (C) *PNMT* in public PCC samples’ cell. A significant positive correlation is observed between MYCN+ PCC cells markers’ score and *NDUFA4L2* expression (R = 0.414, P = 0), while a significant negative correlation is noted between MYCN+ PCC cells markers’ score and *PNMT* expression (R = -0.246, P = 7.31e-168).

(D) A violin plot compares the marker scores of MYCN+ PCC cells across different PCC subtypes. The analysis includes two PCC samples from this study and publicly available dataset samples, representing three metabolism-related PCC subtypes and one kinase-related PCC subtype.

(E) Violin plot showing the expression levels of metabolism-PCC-associated markers (*NDUFA4L2* and *COX4I2*) and kinase-PCC-associated markers (*PNMT* and *RET*) across PCC samples from this study and public datasets.


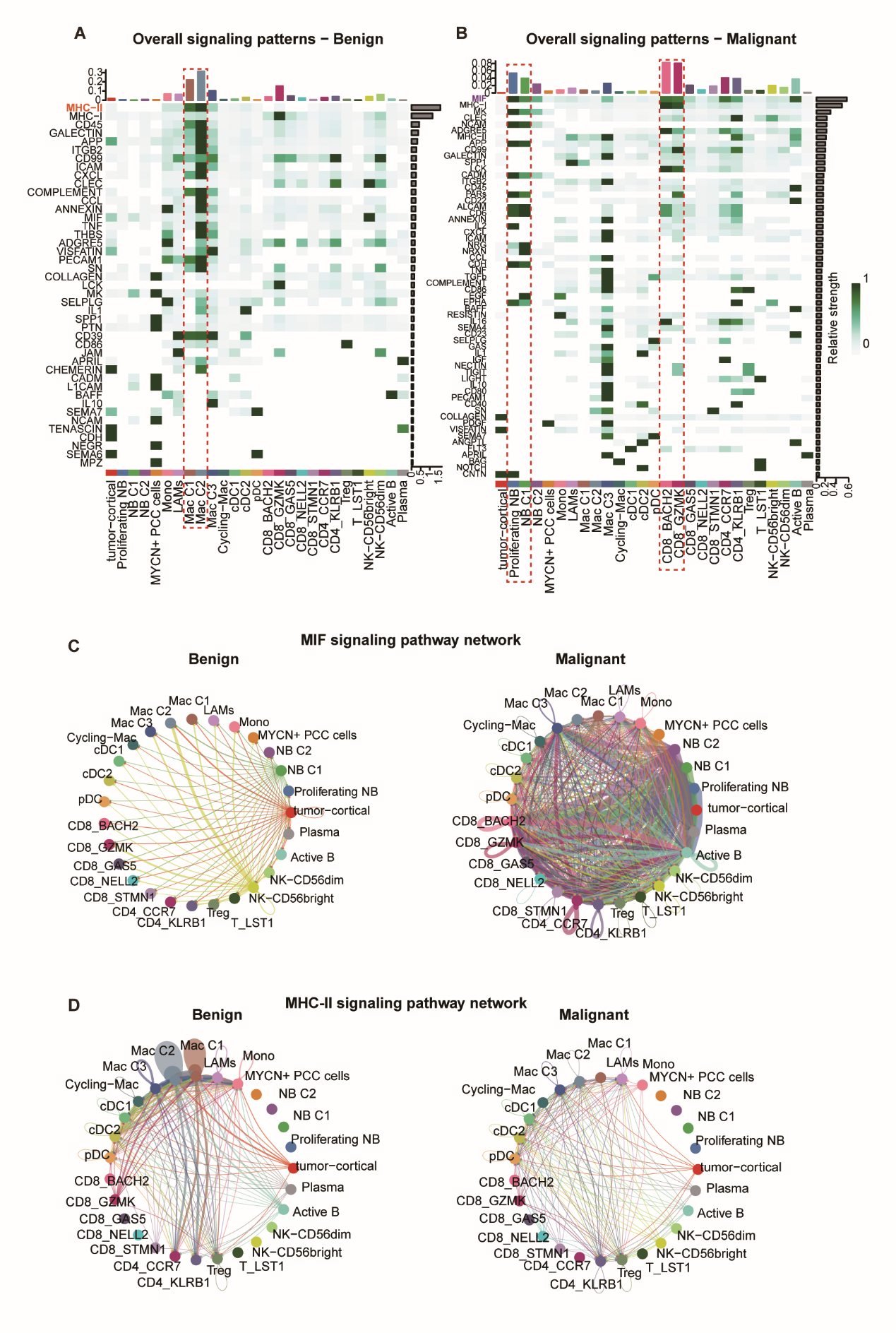


### Figure S8. Tumor-Immune Cell Signaling Networks and MIF Pathway Communication in Benign and Malignant Tumors

(A-B) Heatmaps showing the overall signaling associated with each tumor and immune cell subtype in benign (A) and malignant tumors (B). The relative strength of signaling is indicated by color intensity, with darker green representing stronger communication.

(C-D) Circle plots comparing cell-cell communication mediated by MIF signaling pathway (C) and MHC II signaling pathway (D) in benign (left) and malignant (right) tumors. Edges represent ligand-receptor interactions, and the node size corresponds to the communication strength.

**Table S1.** Clinical and histopathological characteristics of 11 AI cases, Related to Figures 1, S1 and S2

**Table S2.** Basic Information for Single-cell Datasets of 11 AI Samples, 2 Fetal Adrenal Gland Samples and 15 Neuroblastoma Samples, Related to Figures 1, S1 and S2

**Table S3.** Cell Type Markers, Related to Figure 1

**Table S4.** Differentially Expressed Genes and Pathway Analysis (From Normal to Tumor Adrenocortical Cells), Related to Figure 2

**Table S5.** Differentially Expressed Genes (ACA-No vs. FAD; ACA-Conn vs. FAD; ACA-Conn vs. ACA-No), Related to Figure 3

**Table S6.** GO functional analysis (ACA-No vs. FAD; ACA-Conn vs. FAD; ACA-Conn vs. ACA-No), Related to Figure 3

**Table S7.** Medulla Cell Type Markers, Related to Figure 5

**Table S8.** GO functional analysis (MYCN+ PCC cells and proliferating NB cells), Related to Figure 5

**Table S9.** Immune Cell Type Markers, Related to Figure 6
